# Draft Genome Sequence of a Clinical Isolate of Multidrug-Resistant *Mycobacterium tuberculosis* East African Indian Strain OSDD271

**DOI:** 10.1128/genomeA.00541-13

**Published:** 2013-08-01

**Authors:** Shamsudheen Karuthedath Vellarikkal, Ashok Patowary, Meghna Singh, Vinita Periwal, Ajay Vir Singh, Pravin Kumar Singh, Parul Garg, Viswa Mohan Katoch, Kiran Katoch, Pramod Kumar Jangir, Rakesh Sharma, Open Source Drug Discovery Consortium, D. S. Chauhan, Vinod Scaria, Sridhar Sivasubbu

**Affiliations:** Genomics and Molecular Medicine, CSIR Institute of Genomics and Integrative Biology, Delhi, Indiaa; G. N. Ramachandran Knowledge Center for Genome Informatics, CSIR Institute of Genomics and Integrative Biology, Delhi, Indiab; National JALMA Institute of Leprosy and Other Mycobacterial Diseases, Tajganj, Indiac; Energy and Environmental Biotechnology, CSIR Institute of Genomics and Integrative Biology, Delhi, Indiad; CSIR Open Source Drug Discovery Unit, Anusandhan Bhavan, New Delhi, Indiae

## Abstract

We describe the genome sequencing and analysis of a clinical isolate of *Mycobacterium tuberculosis* East African Indian (EAI) strain OSDD271 from India.

## GENOME ANNOUNCEMENT

Tuberculosis has emerged as one of the major public health issues worldwide, accounting for approximately 1.4 million deaths in 2010 alone (1). Over 40% of the global incidence in tuberculosis is accounted for by cases in India and China, with 24% of cases occurring in Africa (1). Closely related species of *Mycobacterium*, named the *Mycobacterium tuberculosis* complex (MTBC), are responsible for the pathogenesis of tuberculosis (2). Clinical isolates of the *Mycobacterium tuberculosis* complex are mainly clustered into distinct lineages based on spoligotype patterns (3). These lineages include the Indo-Oceanic (IO), East African Indian (EAI), East Asian (Beijing), Central Asian (CAS), Euro-American (Haarlem, LAM, T, and X), West African I (AFRI2), and West African lineage II (AFRI1) (4). The CAS and EAI lineages have been found to be distributed across the world. CAS and EAI genotypes, along with the Beijing genotypes, contribute to the major proportion of clinical strains in India (5–8). EAI strains have also been reported as one of the major genotypes in many countries of Southeast Asia and Africa (9). EAI strains have been shown previously to be associated with slow transmissibility in human populations/patients in some parts of the world (10) and along with CAS strains have been suggested to be involved in extrapulmonary tuberculosis in some regions of the world (11, 12). EAI strains have also been important in the study of the evolutionary history of *Mycobacterium tuberculosis*, because they are primitive strains (13).

In this report, we describe the draft genome of a multidrug-resistant clinical isolate of *Mycobacterium tuberculosis* conforming to the EAI spoligotype. The clinical isolate of OSDD271 was obtained from the strain repository maintained at the National JALMA Institute of Leprosy and Other Mycobacterial Diseases and is part of the CSIR Open Source Drug Discovery project open access repository. Spoligotyping was performed and drug sensitivity was evaluated per standard protocols (14–16). Drug sensitivity analysis revealed the isolate to be resistant to rifampin, isoniazid, pyrazinamide, ethambutol, streptomycin, and 4-aminosalicylic acid (PAS). DNA was isolated per standard protocols. The raw sequence data were generated after library preparation on Ion Torrent PGM and Roche 454 platforms according to protocols recommended by the manufacturers. Draft genomes were assembled de novo using CLC Genomics Workbench 6. The assembly resulted in 182 contigs at *N*_50_ values of 37,178 bp and a total assembly of 4,169,405 bp. The scaffolds were ordered using Mauve (17). Automated gene prediction on the draft genomes was performed using the RAST server (18). Analysis revealed that the draft assembly encoded 4,186 genes, including 48 RNA genes.

### Nucleotide sequence accession numbers. 

This whole-genome shotgun project has been deposited at DDBJ/EMBL/GenBank under the accession number AQQC00000000. The version described in this report is version AQQC01000000.

## References

[1] World Health Organization 2011 Global tuberculosis control: WHO report 2011. World Health Organization, Geneva, Switzerland http://www.who.int/tb/publications/global_report/2011/gtbr11_full.pdf

[2] Homolka S Niemann S Russell DG Rohde KH 2010 Functional genetic diversity among *Mycobacterium tuberculosis* complex clinical isolates: delineation of conserved core and lineage-specific transcriptomes during intracellular survival. PLoS Pathog. 6:e1000988. 10.1371/journal.ppat.1000988 20628579 PMC2900310

[3] Brudey K Driscoll JR Rigouts L Prodinger WM Gori A Al-Hajoj SA Allix C Aristimuño L Arora J Baumanis V Binder L Cafrune P Cataldi A Cheong S Diel R Ellermeier C Evans JT Fauville-Dufaux M Ferdinand S Garcia de Viedma D Garzelli C Gazzola L Gomes HM Guttierez MC Hawkey PM van Helden PD Kadival GV Kreiswirth BN Kremer K Kubin M Kulkarni SP Liens B Lillebaek T Ho ML Martin C Martin C Mokrousov I Narvskaïa O Ngeow YF Naumann L Niemann S Parwati I Rahim Z Rasolofo-Razanamparany V Rasolonavalona T Rossetti ML Rüsch-Gerdes S Sajduda A Samper S Shemyakin IG Singh UB Somoskovi A Skuce RA van Soolingen D Streicher EM Suffys PN Tortoli E Tracevska T Vincent V Victor TC Warren RM Yap SF Zaman K Portaels F Rastogi N Sola C 2006 Mycobacterium tuberculosis complex genetic diversity: mining the fourth international spoligotyping database (SpolDB4) for classification, population genetics and epidemiology. BMC Microbiol. 6:23. 10.1186/1471-2180-6-23 16519816 PMC1468417

[4] Gagneux S Small PM 2007 Global phylogeography of *Mycobacterium tuberculosis* and implications for tuberculosis product development. Lancet Infect. Dis. 7:328–337 17448936 10.1016/S1473-3099(07)70108-1

[5] Varma-Basil M Kumar S Arora J Angrup A Zozio T Banavaliker JN Singh UB Rastogi N Bose M 2011 Comparison of spoligotyping, mycobacterial interspersed repetitive units typing and IS6110-RFLP in a study of genotypic diversity of *Mycobacterium tuberculosis* in Delhi, North India. Mem. Inst. Oswaldo Cruz 106:524–535 21894371 10.1590/s0074-02762011000500002

[6] Shanmugam S Selvakumar N Narayanan S 2011 Drug resistance among different genotypes of *Mycobacterium tuberculosis* isolated from patients from Tiruvallur, South India. Infect. Genet. Evol. 11:980–986 21453793 10.1016/j.meegid.2011.03.011

[7] Gutierrez MC Ahmed N Willery E Narayanan S Hasnain SE Chauhan DS Katoch VM Vincent V Locht C Supply P 2006 Predominance of ancestral lineages of *Mycobacterium tuberculosis* in India. Emerg. Infect. Dis. 12:1367–1374 17073085 10.3201/eid1209.050017 PMC3294724

[8] Singh UB Suresh N Bhanu NV Arora J Pant H Sinha S Aggarwal RC Singh S Pande JN Sola C Rastogi N Seth P 2004 Predominant tuberculosis spoligotypes, Delhi, India. Emerg. Infect. Dis. 10:1138–1142 15207071 10.3201/eid1006.030575 PMC3323169

[9] Zhang J Heng S Le Moullec S Refregier G Gicquel B Sola C Guillard B 2011 A first assessment of the genetic diversity of *Mycobacterium tuberculosis* complex in Cambodia. BMC Infect. Dis. 11:1471–2334 10.1186/1471-2334-11-42 PMC3062598 21299851

[10] Albanna AS Reed MB Kotar KV Fallow A McIntosh FA Behr MA Menzies D 2011 Reduced transmissibility of East African Indian strains of *Mycobacterium tuberculosis*. PLoS One 6:19. 10.1371/journal.pone.0025075 PMC3176299 21949856

[11] Svensson E Millet J Lindqvist A Olsson M Ridell M Rastogi N Western Sweden Tuberculosis Epidemiology Study Group 2011 Impact of immigration on tuberculosis epidemiology in a low-incidence country. Clin. Microbiol. Infect. 17:881–887 20825440 10.1111/j.1469-0691.2010.03358.x

[12] Click ES Moonan PK Winston CA Cowan LS Oeltmann JE 2012 Relationship between mycobacterium tuberculosis phylogenetic lineage and clinical site of tuberculosis. Clin. Infect. Dis. 54:211–219 22198989 10.1093/cid/cir788

[13] Sola C Filliol I Legrand E Lesjean S Locht C Supply P Rastogi N 2003 Genotyping of the *Mycobacterium tuberculosis* complex using MIRUs: association with VNTR and spoligotyping for molecular epidemiology and evolutionary genetics. Infect. Genet. Evol. 3:125–133 12809807 10.1016/s1567-1348(03)00011-x

[14] Kamerbeek J Schouls L Kolk A van Agterveld M van Soolingen D Kuijper S Bunschoten A Molhuizen H Shaw R Goyal M van Embden J 1997 Simultaneous detection and strain differentiation of *Mycobacterium tuberculosis* for diagnosis and epidemiology. J. Clin. Microbiol. 35:907–914 9157152 10.1128/jcm.35.4.907-914.1997 PMC229700

[15] National Committee for Clinical Laboratory Standards 2002 Susceptibility testing of mycobacteria, *Nocardia*, and other aerobic actinomycetes. Tentative standard M24-T2, 2nd ed. National Committee for Clinical Laboratory Standards, Wayne, PA 31339680

[16] Canetti G Fox W Khomenko A Mahler HT Menon NK Mitchison DA Rist N Smelev NA 1969 Advances in techniques of testing mycobacterial drug sensitivity, and the use of sensitivity tests in tuberculosis control programmes. Bull. World Health Organ. 41:21–43 5309084 PMC2427409

[17] Darling AE Tritt A Eisen JA Facciotti MT 2011 Mauve assembly metrics. Bioinformatics 27:2756–2757 21810901 10.1093/bioinformatics/btr451 PMC3179657

[18] Aziz RK Bartels D Best AA DeJongh M Disz T Edwards RA Formsma K Gerdes S Glass EM Kubal M Meyer F Olsen GJ Olson R Osterman AL Overbeek RA McNeil LK Paarmann D Paczian T Parrello B Pusch GD Reich C Stevens R Vassieva O Vonstein V Wilke A Zagnitko O 2008 The RAST server: rapid annotations using subsystems technology. BMC Genomics 9:1471–2164 10.1186/1471-2164-9-75 PMC2265698 18261238

